# Artificial Intelligence as a Screening Tool for MRI Evaluation of Normal and Abnormal Medial Meniscus

**DOI:** 10.7759/cureus.82523

**Published:** 2025-04-18

**Authors:** Bondugula Vaishnavi Reddy, Muchchandi Rajashekhar, Koppal Divyashree, Halkude Tejas

**Affiliations:** 1 Radiodiagnosis, Bijapur Lingayat District Educational (BLDE) (Deemed to Be University), Shri B. M. Patil Medical College Hospital and Research Centre, Vijayapura, IND

**Keywords:** artificial intelligence in radiology, deep learning model, magnetic resonance imaging (mri), mask-region convolutional neural networks(rcnn), meniscus

## Abstract

Background

The meniscus is a crucial structure in the knee joint, and its abnormalities are best detected using MRI. However, manual interpretation of MRI scans is time-consuming and prone to inter-observer variability. With advancements in artificial intelligence (AI), deep learning models offer a promising approach to automate evaluation, improving diagnostic accuracy and efficiency.

Objective

This study evaluates the performance of a deep learning-based Mask R-CNN model for the segmentation and classification of the medial meniscus in MRI scans. Unlike prior studies that used bounding box-based segmentation of knee structures, our model utilizes precise polygonal annotations to ensure pixel-wise segmentation limited to the meniscus, allowing accurate abnormality detection.

Methods

We used a dataset of 3,600 sagittal proton density-weighted fat-suppressed (PD-FS) MRI images. The meniscus was manually annotated using the VGG Image Annotator (VIA) tool, focusing on accurate delineation of the meniscus while excluding adjacent anatomical structures. The Mask R-CNN model, with a ResNet-50 backbone and feature pyramid network (FPN), was trained for 50 epochs using a structured dataset. We evaluated performance using metrics such as area under the curve (AUC), segmentation accuracy, sensitivity, and specificity.

Results

The model demonstrated high performance in distinguishing normal and abnormal menisci. It achieved an AUC of 0.992 for normal menisci and 0.963 for abnormal menisci. Segmentation results confirmed precise meniscus delineation, validating the exclusion of non-meniscal regions and improving classification accuracy. Training and validation loss trends showed effective learning without overfitting, supporting the model’s generalization capability.

Conclusion

The Mask R-CNN model provides an accurate AI-assisted tool for segmenting and classifying MRI scans. By using pixel-wise segmentation rather than bounding boxes, our approach minimizes the inclusion of surrounding structures, ensuring more refined and clinically relevant abnormality detection. Focusing solely on the meniscus enables targeted abnormality detection while reducing the radiologists' workload. Future work will focus on multi-center dataset validation and expanding the model to sub-classify meniscal abnormalities for enhanced clinical applicability.

## Introduction

The meniscus, a crucial fibrocartilaginous structure in the knee joint, plays a vital role in load-bearing, shock absorption, and joint stabilization. Both chronic and acute meniscal tears may significantly impair knee function and increase the likelihood of osteoarthritis [1]. The gold standard for identifying meniscal abnormalities is MRI, which is the most advanced non-invasive technique and offers high soft tissue contrast. There is a need to develop automated techniques to improve diagnostic accuracy and efficiency, as MRI scan interpretation is highly specialized, time-consuming, and prone to inter-observer variability [2].

Recent advancements in AI models and deep learning algorithms show considerable potential in medical imaging applications. Convolutional neural networks (CNNs) and their variations, such as mask region-based CNNs (Mask R-CNN), have proven effective in region-based segmentation and classification tasks in radiology [3]. Automated meniscus segmentation can be achieved using Mask R-CNN with manually annotated data. In addition to reducing radiologists’ workloads and aiding in more accurate diagnoses, these AI models can process large datasets and quickly detect subtle pathological changes [4].

This study aimed to evaluate deep learning-based models for MRI-based evaluation of the medial meniscus. The goal is to develop an AI-based screening tool that can accurately distinguish between normal and abnormal menisci. Several studies have explored deep learning models for meniscal tear detection. For example, Bien N et al. (2018) used a deep CNN model to classify meniscal tears, achieving an area under the curve (AUC) of 0.91. Similarly, Couteaux V et al. (2019) implemented U-Net-based segmentation for meniscal abnormalities with promising results. Most existing models focus solely on classification, whereas our study integrates both classification and region-based segmentation using , which enhances localization accuracy by focusing on targeted segmentation of the meniscus.

To improve generalisability, we trained the proposed deep learning model with a large dataset of pre-processed MRI images. However, we did not use any data augmentation techniques, as the dataset was sufficiently extensive. We used metrics such as AUC, classification accuracy, and segmentation productivity to assess the model’s performance. These results provide valuable insights into the potential of AI in musculoskeletal imaging and will guide its eventual implementation in clinical settings.

## Materials and methods

Data collection

This study involved a retrospective and prospective analysis of MRI cross-sections of the medial meniscus of the knee in individuals from Vijayapura. The dataset was acquired using a GE SIGNA Explorer 1.5 Tesla MRI Scanner. MRI images of the medial meniscus were obtained in sagittal sections using fat-suppressed proton density-weighted (FS-PDW) sequences. Imaging parameters include TR 2000-4000 ms, TE 30-50 ms, slice thickness ~3 mm, FOV 150-180 mm, and matrix 256x256. Sagittal slices were planned perpendicular to the femoral condyles to optimize visualization of the medial meniscus. This sequence is preferred for deep learning models due to optimal tissue contrast between the meniscus, cartilage, and soft tissues and enhanced visibility of meniscal abnormalities. Patient data was collected after obtaining necessary ethical approvals and anonymization. The dataset consisted of 3,600 MRI images from 800 patients, excluding images with motion artifacts and post-operative knee surgery scans. The images were stored in DICOM format and retrieved from the Picture Archiving and Communication System (PACS). The dataset was split into training data of 2,520 (70%) images, validation data of 360 (10%) images, and testing data of 720 (20%) images.

Inclusion and exclusion criteria

Inclusion Criteria

MRI image type: Sagittal fat-suppressed proton density-weighted (FS-PDW) MRI images

Patient demographics: Images from individuals of any age

Data verification: Images must be verified during pre-processing to ensure they meet the study's standards

Exclusion Criteria

MRI images with motion artifacts or magnetic artifacts, images where the visibility of cartilage is incomplete and MRI images taken from patients who have undergone knee surgery.

The main objective of the challenge is to build a high-performance deep learning-based artificial intelligence model utilizing a modified Mask R-CNN for the detection and segmentation of menisci. The model is designed to differentiate between normal and abnormal menisci, including post-traumatic and degenerative cases. The annotations are limited to the meniscus without encompassing surrounding structures, so the segmentation approach is targeted rather than fully automatic.

Convolutional neural network

Mask R-CNN is an advanced deep learning architecture and one of the most recent models for object detection and segmentation. It was first introduced by Kaiming He et al. in 2017, building upon their earlier Faster R-CNN model from 2015. Mask R-CNN extends the Faster R-CNN architecture by adding an extra branch for predicting segmentation masks on each region of interest, thereby enabling high-quality segmentation results. The architecture consists of: Backbone Network: A CNN (e.g., ResNet-50 or ResNet-101) that extracts feature maps from the input image [5]; Region Proposal Network (RPN): identifies candidate object regions [6]; RoI Align: accurately maps proposed regions to the feature map, preserving spatial details [7]; and multi-branch outputs: Includes bounding box detection for object localization, class label prediction for classification, and mask prediction for pixel-wise segmentation.

Mask R-CNN is widely used for object detection and segmentation and has applications in medical imaging and autonomous systems. It includes a contracting path (encoder) to capture high-level features through convolutional and pooling layers, followed by a bottleneck layer to gather deeper representations. The expanding path (decoder) restores spatial quality using transposed convolution and upsampling layers. Skip connections between the encoder and decoder preserve fine-grained information, and the final segmentation map is produced via a convolutional layer with a softmax activation [7]. Mask R-CNN is particularly advantageous for medical image segmentation, outperforming many conventional models [8].

**Figure 1:**
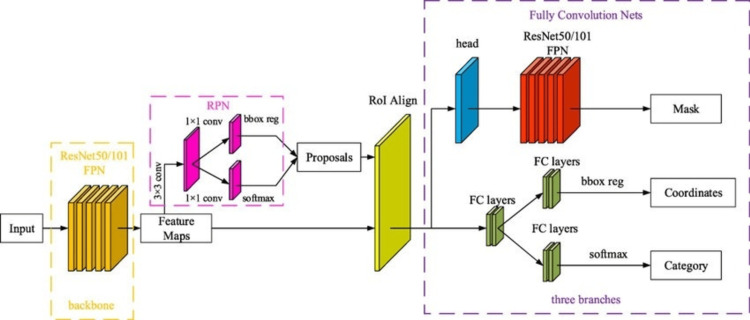
Mask R-CNN architecture. Mask R-CNN: Mask region-based convolutional neural network. Adapted from: Obstacle detection in dangerous railway track areas by a convolutional neural network – Scientific Figure on ResearchGate. Available from: https://www.researchgate.net/figure/Main-network-structure-of-Mask-R-CNN_fig2_352679679 (accessed March 26, 2025). Licence: https://creativecommons.org/licenses/by/4.0/

Similarly, the Region-CNN architecture follows an encoder-decoder framework. The encoder network uses convolution and pooling layers with ReLU activation to extract image features. Pooling indices track the positions of max-pooled elements to aid in upsampling, while the decoder reconstructs spatial features. Skip connections improve segmentation performance by preserving spatial information across multiple resolution levels. The final segmentation map is generated using a softmax activation function [9]. This architecture is known for its efficiency, scalability, and successful application in biomedical image segmentation, road segmentation, and object detection [10]. Figure 1 shows the Mask R-CNN architecture [11].

Training

**Figure 2:**
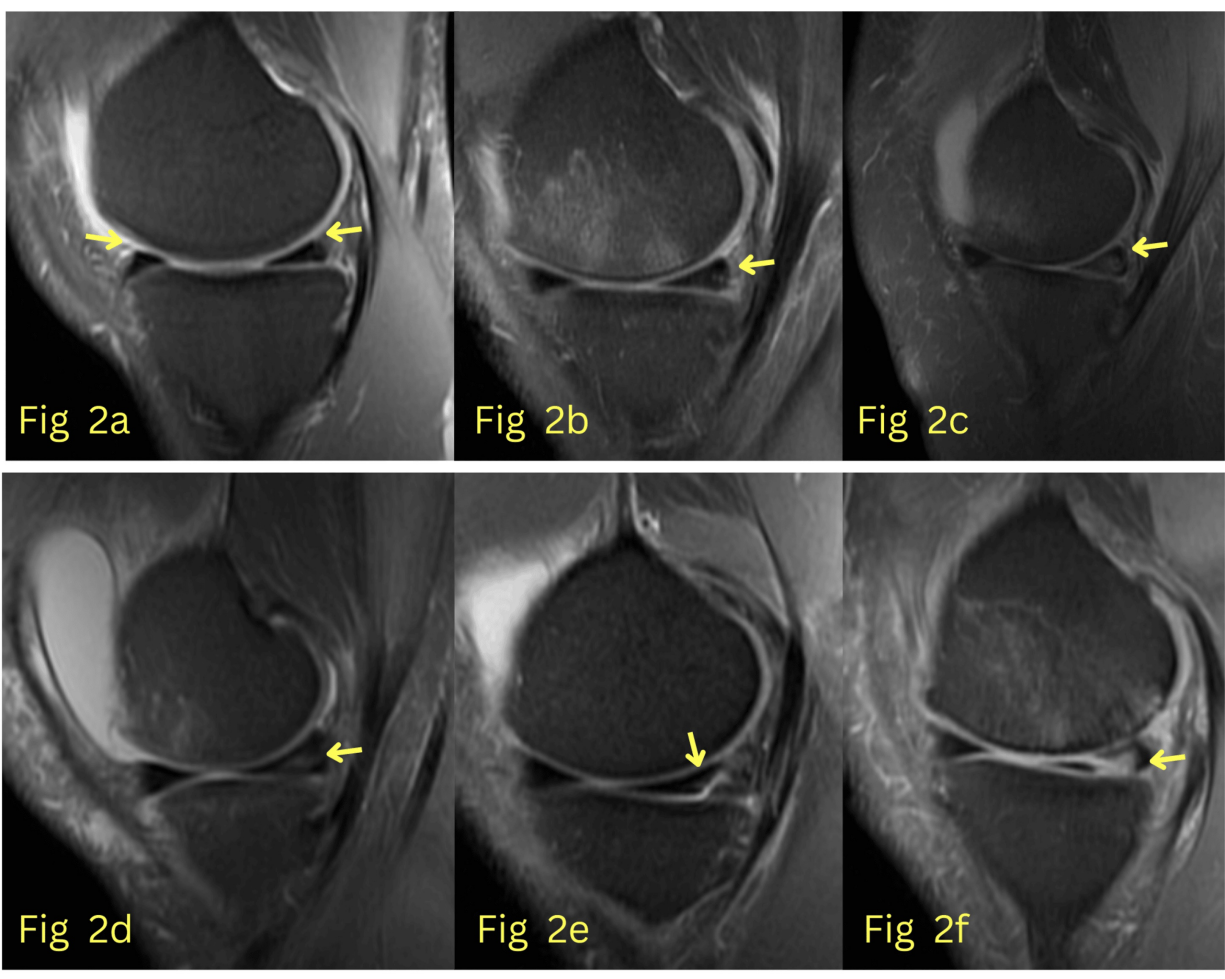
(a) Normal anterior and posterior horn of the medial meniscus; (b) Grade I signal intensity change in the posterior horn; (c) Wedge-shaped Grade II signal intensity change in the posterior horn; (d) Linear Grade II signal intensity change; (e) and (f) Grade III signal intensity changes.

**Figure 3:**
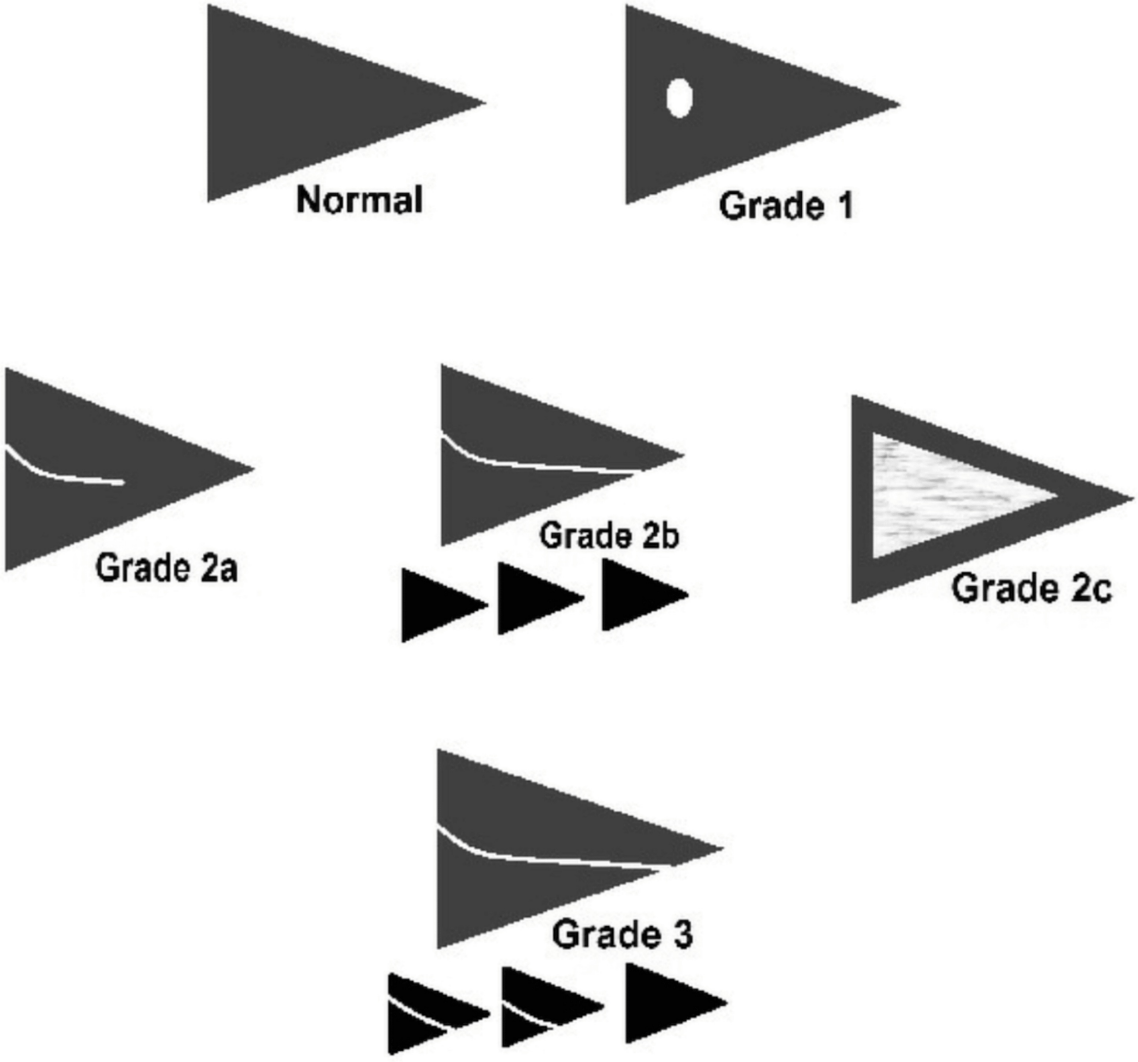
MRI grading system of abnormal meniscal signal intensity. Adapted from: Amer M et al. [12] License: https://creativecommons.org/licenses/by-nc-sa/3.0/

The training dataset was composed of 2,520 images with a matrix resolution of 256 × 256. The dataset consisted of both normal and abnormal medial meniscus images (all images with Grade I, II, and III signal intensity changes according to the MRI grading system for abnormal meniscal signal intensity were included). Figure 2 shows the training images from the dataset, and Figure 3 illustrates the MRI grading system for abnormal meniscal signal intensity [12].

Grade I signal intensity changes are very small, and even diagnosing them is difficult due to signal interference from surrounding structures or joint effusions observed in both degenerative and post-traumatic changes. However, the model's accuracy can be improved by using larger datasets. Manual annotation was systematically and carefully performed by experienced radiologists using the VGG Image Annotator (VIA) to acquire pixel-wise segmentation masks outlining the meniscus area in the MRI images.

**Figure 4:**
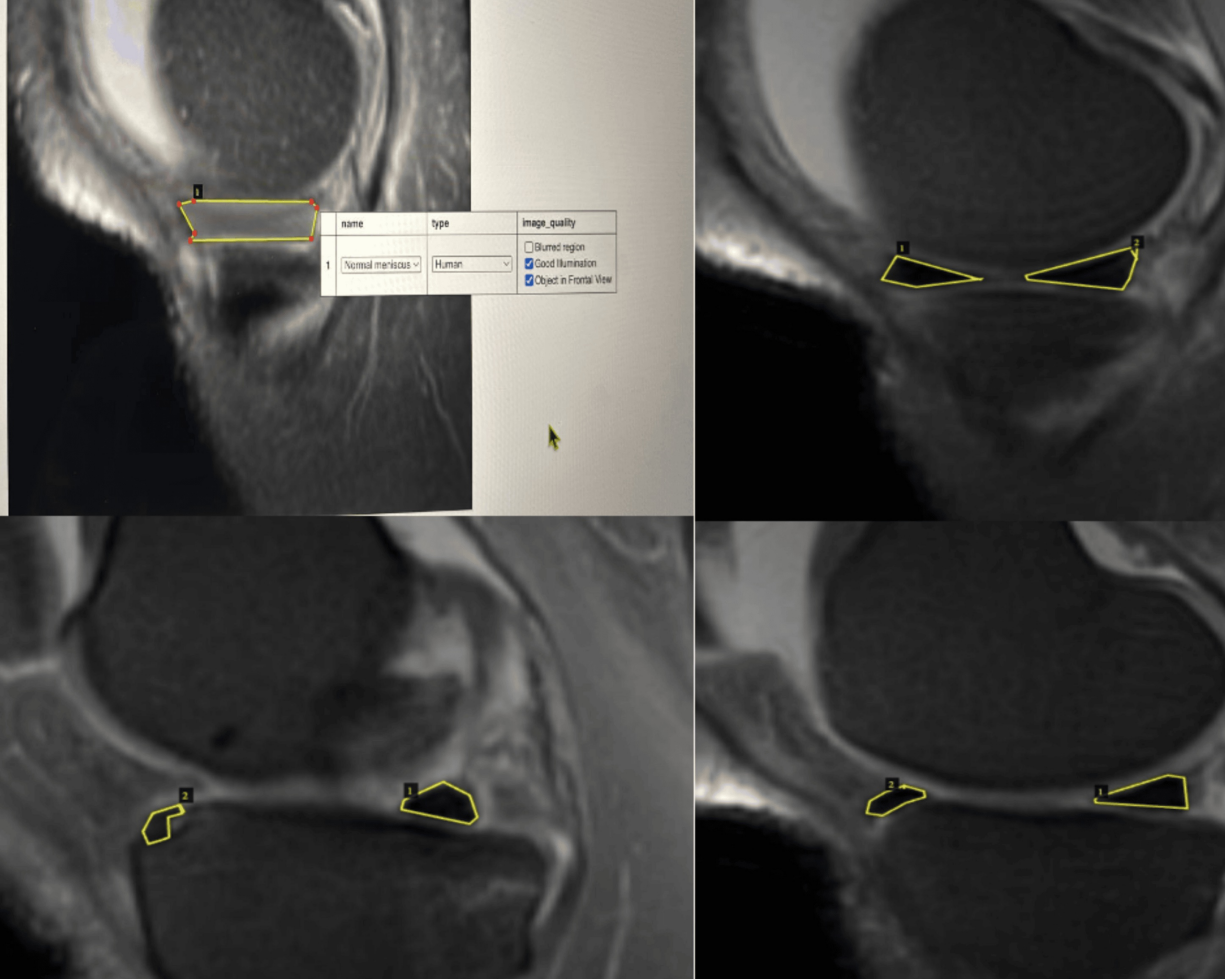
Annotated images using the VGG Image Annotator.

Region-based manual annotation involved the precise labeling of the meniscus borders to produce high-quality ground truth masks that accurately reflect the anatomical structures. The polygonal segmentation approach, carried out using the VIA tool, enabled a flexible method of polygonal marking. This was particularly advantageous for capturing the irregular and complex nature of the meniscus structure. Figure 4 shows the annotated images using the VGG Image Annotator. The medial meniscus was annotated with two classes: Class 1 - Normal medial meniscus; Class 2 - Abnormal medial meniscus.

**Figure 5:**
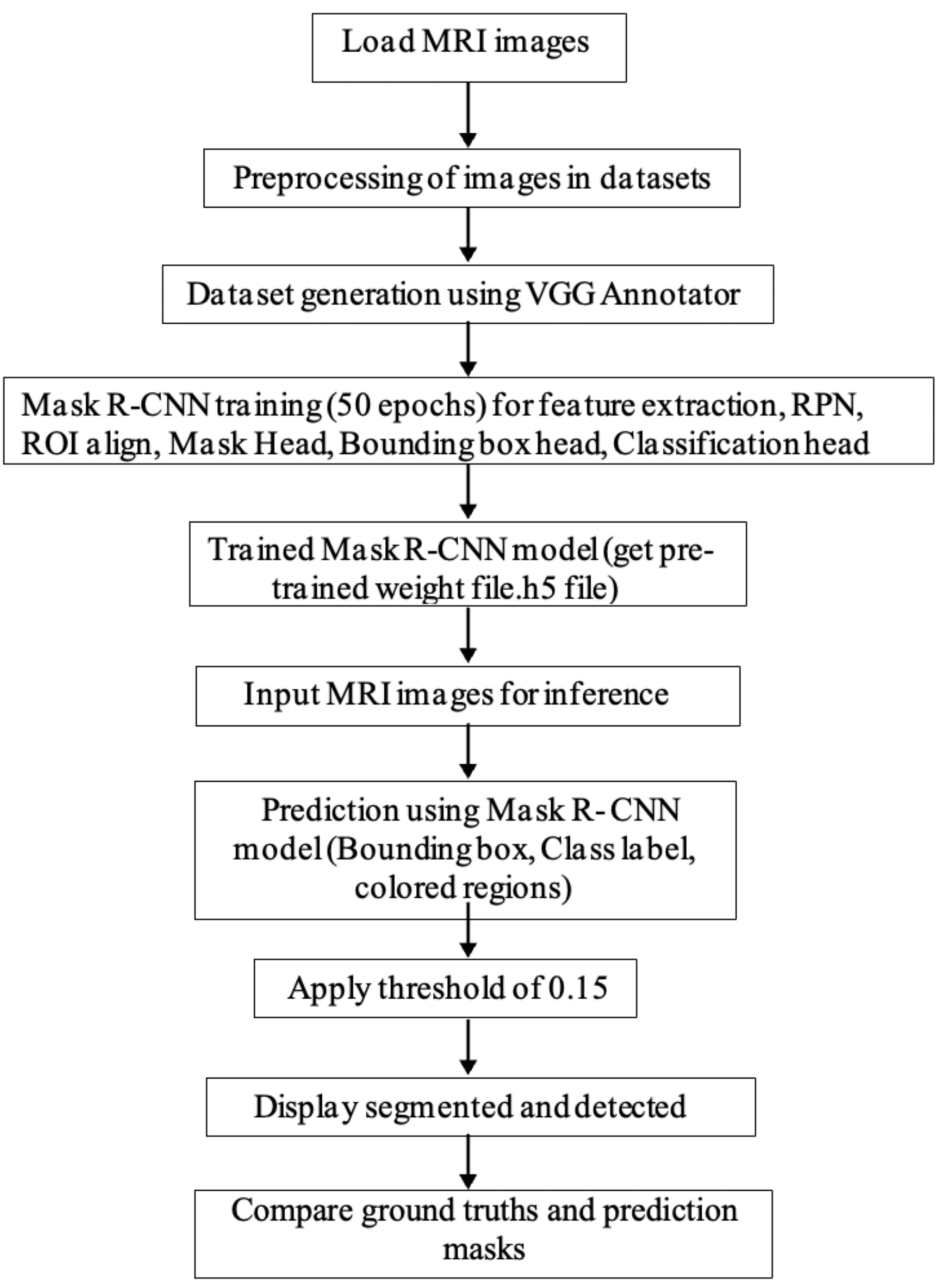
Methodology of the model.

Annotations were saved in JSON format and mapped to corresponding images. Since Mask R-CNN requires a structured dataset with image-mask pairings, a preprocessing step was implemented to convert the JSON annotations into a format compatible with Mask R-CNN. Figure 5 shows the methodology of the model.

The model was trained using Mask R-CNN with a ResNet-50 backbone and a Feature Pyramid Network (FPN) for enhanced multi-scale feature extraction, which has been shown to improve object detection and post-traumatic performance in medical imaging applications [7,13]. A learning rate of 0.001 was selected to balance convergence speed and stability. A momentum of 0.9 was used to accelerate training and reduce oscillations, as recommended in previous deep learning studies [14]. The batch size was set to 8 to optimize memory utilization while maintaining stable training dynamics, particularly given the high-resolution nature of MRI images. The model was trained for 50 epochs, as performance gains beyond this point were marginal, with an increased risk of overfitting. The entire training process, executed on a high-performance GPU, was completed in 14 hours, demonstrating computational efficiency while maintaining high accuracy. These hyperparameter choices were refined through iterative fine-tuning to ensure that the Mask R-CNN model effectively segmented the medial meniscus while preventing overfitting and supporting generalization.

Validation

Following the completion of model training, validation was conducted to assess generalization performance. A 10% validation set (360 images) was used to evaluate the model at the end of each epoch, ensuring that the learned features were not overfitting to the training data. Although some deep learning applications allocate 15-20% for validation, we determined that a 10% split provided an optimal balance, allowing for effective learning while preserving a larger training set. Given the relatively large dataset, this distribution ensured robust validation without significantly reducing the amount of training data. At the end of each epoch, the training model was evaluated using the validation dataset to monitor model convergence and generalization. The training and validation loss curves (see Figure 10 in the Results section) were monitored at each epoch to assess model performance and detect signs of overfitting or underfitting.

Precision and recall

Precision and recall are crucial evaluation metrics for assessing model performance in medical image analysis. Precision measures the proportion of correctly identified positive cases among all predicted positives, while recall quantifies the model’s ability to detect all positive cases. In the context of meniscal tear detection, a high-recall algorithm would classify most menisci as torn, ensuring fewer false negatives but potentially increasing false positives. Conversely, a high-precision algorithm would only classify a meniscus as torn when the confidence level is extremely high, reducing false positives but potentially missing actual tears. For clinical applications, especially when used as a decision-support tool for radiologists, prioritizing recall ensures that all potential tears are detected, allowing doctors to filter out false positives later. To balance these trade-offs, we set a classification threshold of 0.15 in the torn versus standard classification step. A lower threshold favors recall by capturing more potential cases, while a higher threshold increases precision by reducing false alarms. By optimizing this parameter, our model achieves a balance between sensitivity and specificity, ensuring clinical applicability while minimizing diagnostic errors.

## Results

Quantitative evaluation

**Figure 6:**
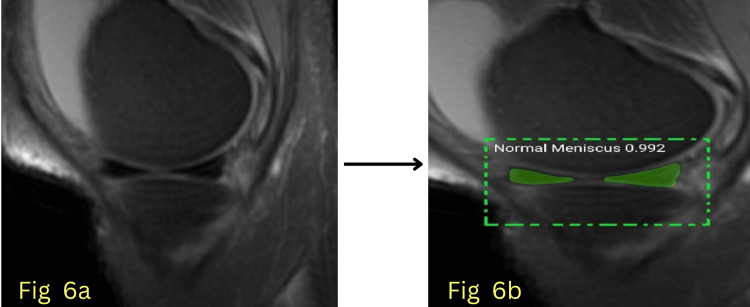
Fig 6a is a input image, Fig 6b shows segmented image of normal anterior and posterior horn

**Figure 7:**
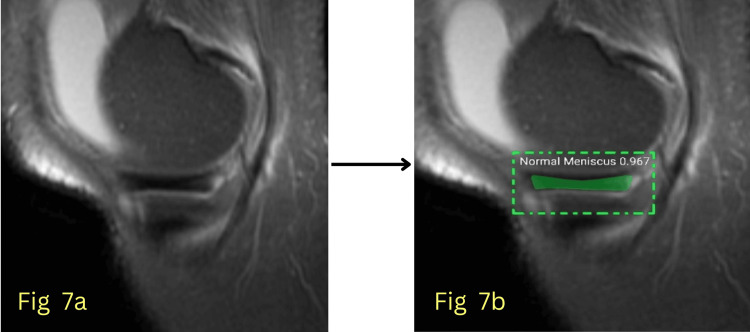
(a) Input MRI image; (b) Segmented image showing the normal body of the meniscus.

**Figure 8:**
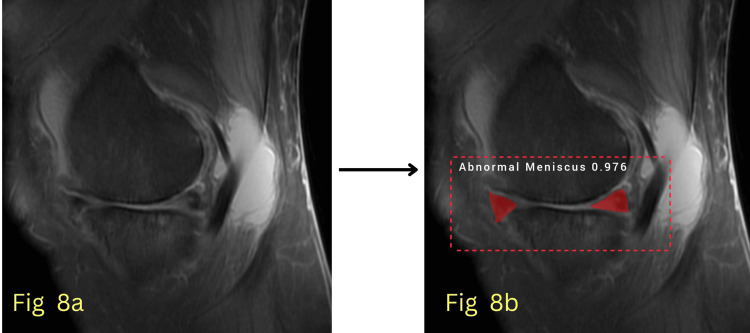
(a) Input MRI image; (b) Segmented image showing abnormal anterior and posterior horns of the meniscus.

**Figure 9:**
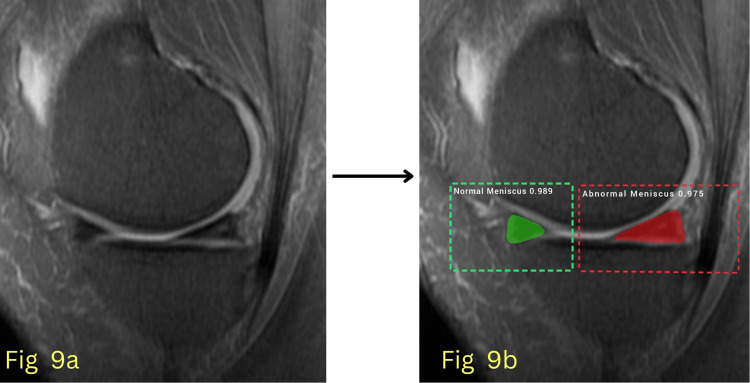
(a) Input MRI image; (b) Segmented image showing a normal anterior horn and an abnormal posterior horn of the meniscus.

The model’s performance was evaluated using key quantitative metrics, primarily the AUC, to assess its ability to differentiate between normal and abnormal menisci with high precision. Additionally, segmentation accuracy, sensitivity, and specificity were analyzed to comprehensively evaluate the model's effectiveness in clinical settings. The model achieved an AUC of 0.992 for normal meniscus detection during testing, indicating near-perfect classification accuracy, and an AUC of 0.963 for abnormal meniscus detection, showing robust performance in identifying meniscal pathology. Figures 6-7 show detected normal meniscus highlighted in green, demonstrating precisely targeted segmentation; Figure 8 shows detected abnormal meniscus highlighted in red, with accurate region localization; and Figure 9 shows detection of a normal anterior horn and an abnormal posterior horn of the meniscus.

Training and validation loss graph

Training and Validation Loss Curve

**Figure 10:**
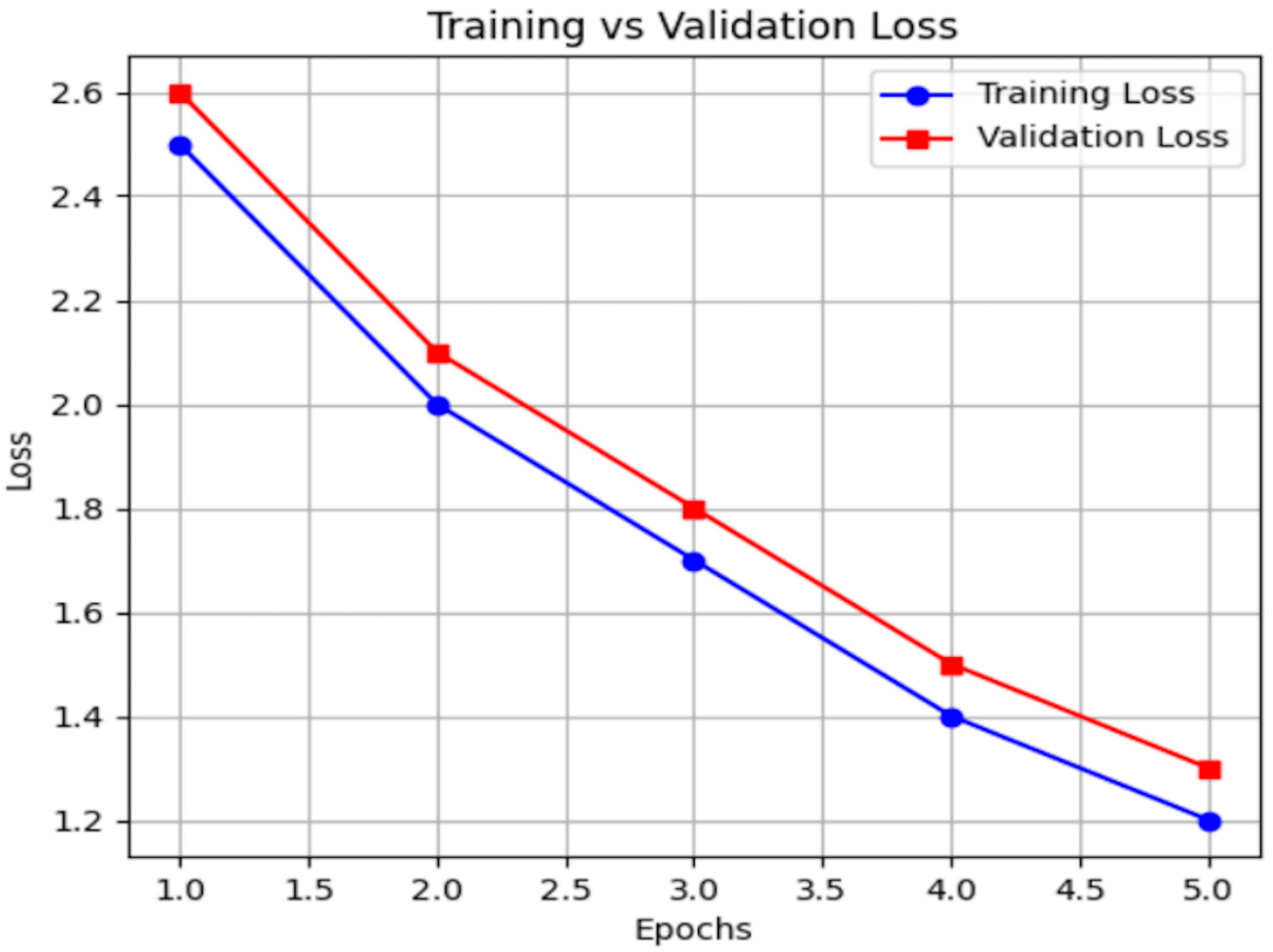
Training and validation loss curve.

This shows the training loss (blue line with circular markers) and validation loss (red line with square markers) over five epochs. The trend indicates that both training and validation losses decrease consistently, suggesting that the model is learning effectively. The absence of divergence between the curves indicates no immediate signs of overfitting. As expected, training loss is slightly lower than validation loss at each epoch, and the small, consistent gap between them indicates good generalization. Even with continued monitoring over additional epochs, there was no significant plateauing of the validation loss (Figure 10).

Segmentation analysis

To complement our classification results, a region-based segmentation approach was employed. The extracted menisci are represented in grayscale images, allowing for more detailed structural analysis. The segmentation results were validated using Intersection over Union (IoU) and Dice Coefficient metrics, which provide a quantitative measure of the overlap between predicted and ground truth segmentations.

Intersection Over Union (IoU)

IoU measures the overlap between the predicted segmentation mask and the ground truth mask:

$&quot;IoU= \frac{(|A\cup B|)}{(|A\cap B|)}&quot;$

Where A is the predicted segmentation region, B is the ground truth segmentation region, $&quot;A\cap B&quot;$ is the intersection (overlap) between the two, and "$A\cup B&quot;$ is the union (total area covered by both). The IoU score ranges between (0, 1), with higher values indicating better performance.

Dice Coefficient (Dice Similarity Coefficient, DSC)

The Dice Coefficient measures the similarity between two sets (predicted and ground truth masks). The range should be between (0, 1) (higher is better).

$&quot;DSC=\frac{(2|A\cap B|)}{|A|+|B|}&quot;$

**Figure 11:**
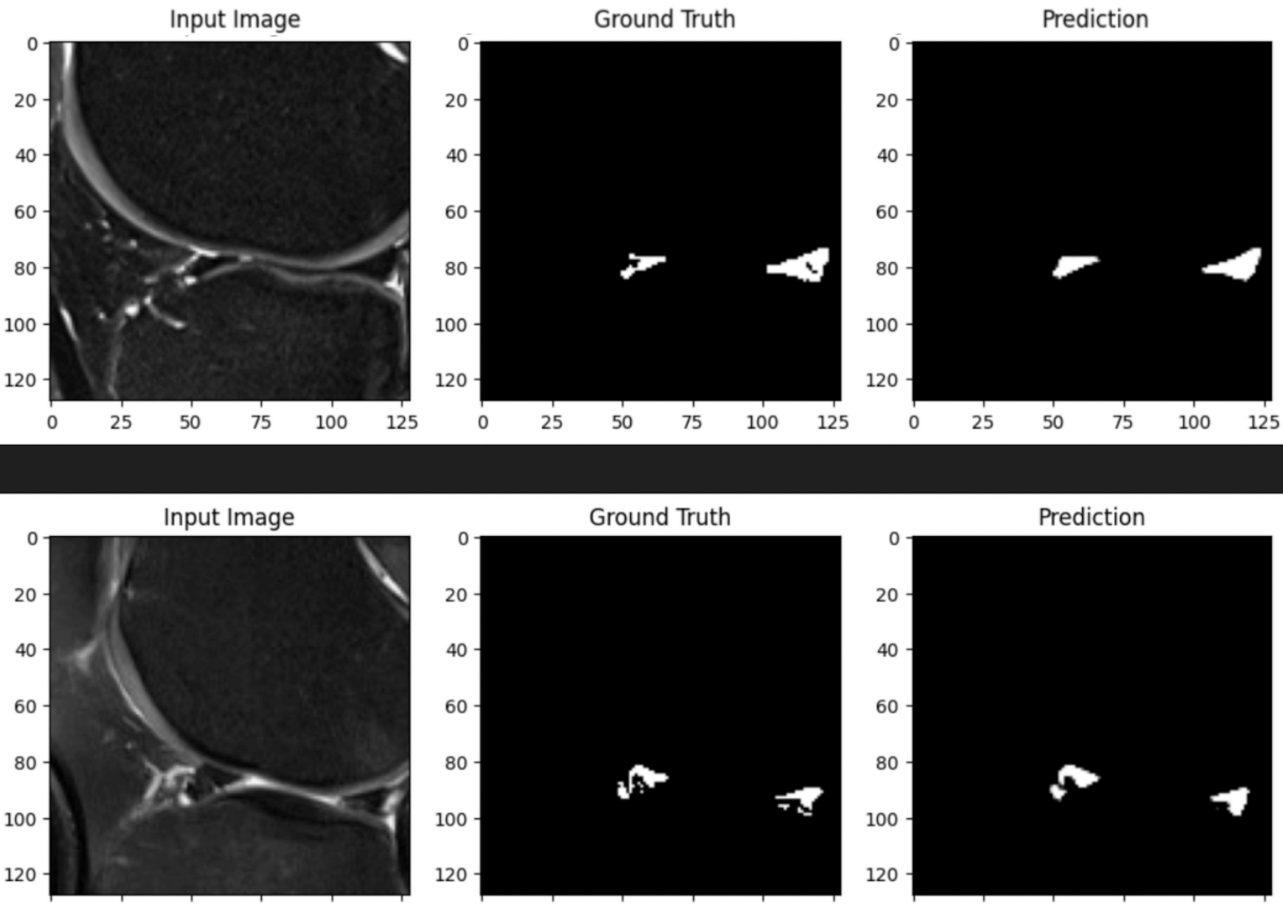
Illustrates the segmentation performance of the proposed model for detecting the medial meniscus in knee MRI scans. The left column presents the original input MRI images, the middle column shows the corresponding ground truth segmentation masks, and the right column displays the model’s predictions. The ground truth masks highlight the medial meniscus, while the prediction masks demonstrate the model’s ability to accurately segment the structure. The results indicate a strong correlation between the predicted and actual meniscus regions, validating the model’s effectiveness in identifying and segmenting the medial meniscus.

Figure 11 shows a sample of the segmentation results in the meniscus regions compared with the ground truth.

Taking the average across all 720 images, the IoU and Dice scores were calculated to provide a comprehensive evaluation of segmentation performance: These metrics serve as a direct assessment of how well the model predictions align with the actual meniscus structures.

$&quot;Mean IoU = \frac{1}{720}\sum_{^{i=1}}^{720}IoU_{i}&quot;$

$&quot;Mean Dice =\frac{1}{720}\sum_{^{i=1}}^{720}Dice_{i}&quot;$

The segmentation was evaluated using the mean Dice coefficient, and the model achieved a Dice score of 0.817. The results obtained in this study validate the performance of the Mask R-CNN model in accurately segmenting and classifying medial menisci on MRI scans using color-coded visualization (red/green). At the same time, the segmentation outputs are presented in grayscale for clarity and better contrast in binary masks. The high AUC values for both normal and abnormal menisci, along with precise region-based segmentation, highlight the model’s effectiveness in automated musculoskeletal imaging. These findings provide a strong foundation for further discussion on clinical applicability, potential integration into radiology workflows, and the need for multicenter validation to enhance generalizability.

## Discussion

The deep learning model developed in this study demonstrated excellent performance in screening for normal and abnormal medial menisci. One limitation is that all images were sourced from a single institution, which may limit the model’s generalizability. Variations in scanner type, imaging protocols, and patient demographics across multiple institutions could impact performance. Further studies should include multi-center datasets to improve the model’s robustness and ensure broader applicability [2]. Additionally, incorporating different MRI sequences and parameters could further enhance generalizability [5]. Another limitation is that our model does not subclassify abnormal menisci into specific pathology types such as tears, degenerative changes, or other meniscal disorders. Since these conditions have different clinical implications, future model iterations should incorporate finer classifications. The ability to differentiate between various abnormalities would significantly enhance the clinical relevance and decision-making process for radiologists and orthopedic specialists [3,4].

Integrating AI into real-world clinical practice requires seamless implementation into Picture Archiving and Communication Systems (PACS). This will facilitate automated pre-processing of images, real-time analysis, and structured reporting. Studies suggest that AI models perform best when integrated into clinical workflows, reducing manual workload while maintaining diagnostic accuracy [4]. Additionally, explainability remains a key challenge, as clinicians may hesitate to trust black-box models without a clear explanation of their decision-making processes. Using explainable AI techniques, such as Gradient-weighted Class Activation Mapping (Grad-CAM) and attention mechanisms, can improve transparency and foster greater trust among healthcare professionals [7]. While our study primarily focused on segmentation and classification of the medial meniscus using deep learning, model interpretability remains crucial for clinical adoption. Techniques such as Grad-CAM can be utilized in future work to visualize which regions of MRI scans influence the model’s predictions. These methods can enhance trust in AI-assisted diagnosis by ensuring that the model focuses on clinically relevant features rather than extraneous regions [7,8].

In summary, our findings support the potential of AI-driven solutions for screening medial meniscus abnormalities in MRI scans. However, improving dataset diversity, refining classification capabilities, and ensuring seamless integration into clinical workflows will be essential steps toward real-world application. Future research should address these challenges to maximize the impact of AI in musculoskeletal imaging.

## Conclusions

This study highlights the potential of deep learning in automating the detection of medial meniscus abnormalities in MRI scans. The Mask R-CNN model achieved high accuracy in both segmentation and classification, ensuring precise abnormality detection. Future work should focus on external validation, seamless PACS integration, and sub-classification of pathologies to enhance clinical utility. A multidisciplinary approach combining AI and clinical expertise is essential to optimize musculoskeletal imaging, improve diagnostic efficiency, and enhance patient outcomes.
